# Editorial: Artificial intelligence and the future of work: humans in control

**DOI:** 10.3389/frai.2024.1378893

**Published:** 2024-03-13

**Authors:** Ekkehard Ernst, Janine Berg, Phoebe V. Moore

**Affiliations:** ^1^International Labour Organization, Geneva, Switzerland; ^2^School of Business, Faculty of Social Sciences, University of Essex, Colchester, East of England, United Kingdom

**Keywords:** artificial intelligence, world of work, occupational safety and health (OSH), employment, wages, recruitment, ethics, productivity

Latest developments around artificial intelligence (AI) have triggered excitement about the potential to replace and complement human activities while also raising concerns about possible risks to society. Dramatic effects are specifically being felt in the world of work, including jobs, wages and working conditions but also recruitment, performance monitoring, and dismissal. So far, research in this area has focused predominantly on the potential of AI for job gains and losses. Other aspects of its transformative dynamics have received less attention, however. In particular, the impact of AI on job quality, average hours worked, mobility, or labor relations between employers and workers are often overlooked. Moreover, society-wide effects triggered by AI, including its rising environmental burden, need to be reassessed. To address these issues, this Research Topic includes nine exciting contributions that shed light on a broader range of issues that AI technologies might bring to the world of work.

To set the stage for the overall effect of AI on employment in 23 OECD countries, in our special edition, Georgieff and Hyee present research using an adapted AI occupational impact measure. The authors do not find that AI exposure affects employment growth in their sample. However, occupations where computer use is high see faster employment growth when exposed to AI. In contrast, occupations with low computer use see a decline in average hours work (yet not in employment) when exposed to AI, suggesting a distributional impact of AI rather than one on the overall number of jobs.

Whether digital technological technologies improve or worsen wages for employees remains a hotly debated topic. Fossen et al. argue that it depends on the specific application considered. Whereas, software and industrial robots seem to be associated with wage decreases, suggesting job displacement, innovations in AI are associated with wage increases, pointing toward positive productivity effects, at least as far as the labor market in the United States is concerned.

How can the income and wealth disparities that are brought about by AI be addressed? Merola looks at the various proposals that have been brought forward in recent years to address the differential effects of AI on labor markets. She discusses pros and cons of various proposals, including a robot tax, digital taxation, share price taxation, or – alternatively – wage subsidies for low-income earners and assesses their potential impact on employment growth, inequality and innovation.

Besides the impact of AI on the number of jobs or their distribution, AI will also affect working conditions for those in employment. Using a representative business survey for Germany, Warning et al. demonstrate how occupations with a high share of routine cognitive activities exposed to AI are associated with higher demand for flexibility, including employee self-organization and time management. Moreover, such worsening of working conditions predominantly affects older workers and women in the labor market.

Concerns about implications of AI for occupational health and safety (OSH) abound. Niehaus et al. report results from a large-scale study of German workers on the impact of AI on job autonomy and psychological occupational stress. The authors highlight that AI is often being used to increase autonomy of supervisory functions while lowering control for job execution. This is likely to increase work-related stress over and above possible concerns for job or earnings loss.

AI is also a tool that can be used by HR managers to improve functional mobility and ultimately employees' job satisfaction, given that internal mobility risks deteriorating job satisfaction if it increases stress to the detriment of personal life. Bossi et al. analyze various approaches using AI to help better manage internal mobility schemes with a view to improving future job satisfaction. The authors analyse alternative statistical models and compare different methods in supporting predictive Human Resources analytics.

AI has society-wide implications not only for employment and wages but also on the use of energy and its potential for increasing productivity. Ernst argues that taken together and considering the current path of technological development, AI gives rise to a trilemma, making it impossible to achieve high productivity growth, low inequality, and reduced energy consumption simultaneously. Instead, he argues, a new technological paradigm is needed to orient AI applications toward those areas where social returns are particularly high, such as in mobility and waste management, clean energy, and natural capital solutions.

Beyond automating certain tasks at individual workplaces, AI is also transforming managerial control. Woodcock analyses in detail how AI affects the work of managers, based on a case study of AI's use in call centers. He shows how the introduction of new surveillance tools based on AI are being contested by call center employees and how this shapes the extent and incidence of such tools for managerial purposes.

Society-wide implications of AI have often met with calls for “ethical Artificial Intelligence.” Cole et al. conceptualize these calls and question their efficacy in sufficiently addressing the resulting societal challenges given their mostly narrow political framework focused on privacy, transparency and non-discrimination. In response, the authors identify a set of principles to facilitate fairer working conditions with AI, focusing on operationalizable processes that effectively help address the potential risks and harms resulting from AI in the workplace.

The collection of papers brought together in this Research Topic offer new insights into the multi-faceted and society-wide implications that AI is likely to bring to the world of work. Our ambition was to demonstrate how current technological changes will cause a wide-ranging transformation that goes beyond headline indicators such as the number of potential job losses. We hope that these papers can contribute to a wider discussion both among researchers and policy makers of the multi-faceted effects of AI on the world of work, and thus for the need to better understand – and find appropriate responses to – the multitude of ongoing changes in the world of work.

## Author contributions

EE: Writing – original draft. JB: Writing – review & editing. PM: Writing – review & editing.

